# Cardiomyopathy-Related Mutations in Cardiac Troponin C, L29Q and G159D, Have Divergent Effects on Rat Cardiac Myofiber Contractile Dynamics

**DOI:** 10.1155/2012/824068

**Published:** 2012-09-12

**Authors:** Sampath K. Gollapudi, Murali Chandra

**Affiliations:** Department of Veterinary and Comparative Anatomy, Pharmacology, and Physiology (VCAPP), Washington State University, Pullman, WA 99164-6520, USA

## Abstract

Previous studies of cardiomyopathy-related mutations in cardiac troponin C (cTnC)—L29Q and G159D—have shown diverse findings. The link between such mutant effects and their divergent impact on cardiac phenotypes has remained elusive due to lack of studies on contractile dynamics. We hypothesized that a cTnC mutant-induced change in the thin filament will affect global myofilament mechanodynamics because of the interactions of thin filament kinetics with both Ca^2+^ binding and crossbridge (XB) cycling kinetics. We measured pCa-tension relationship and contractile dynamics in detergent-skinned rat cardiac papillary muscle fibers reconstituted with the recombinant wild-type rat cTnC (cTnC_WT_), cTnC_L29Q_, and cTnC_G159D_ mutants. cTnC_L29Q_ fibers demonstrated a significant decrease in Ca^2+^ sensitivity, but cTnC_G159D_ fibers did not. Both mutants had no effect on Ca^2+^-activated maximal tension. The rate of XB recruitment dynamics increased in cTnC_L29Q_ (26%) and cTnC_G159D_ (25%) fibers. The rate of XB distortion dynamics increased in cTnC_G159D_ fibers (15%). Thus, the cTnC_L29Q_ mutant modulates the equilibrium between the non-cycling and cycling pool of XB by affecting the *on/off* kinetics of the regulatory units (Tropomyosin-Troponin); whereas, the cTnC_G159D_ mutant increases XB cycling rate. Different effects on contractile dynamics may offer clue regarding how cTnC_L29Q_ and cTnC_G159D_ cause divergent effects on cardiac phenotypes.

## 1. Introduction

The presumptive conclusion drawn from the heterogenic nature of human cardiomyopathy suggests a link between the type of mutation and the nature of pathological remodeling of the heart. A growing number of mutations in human cardiac troponin C (cTnC), associated with either hypertrophic cardiomyopathy (HCM) or dilated cardiomyopathy (DCM), makes it a prominent target gene for functional characterization. Thus far, 5 mutations in cTnC are found to be associated with HCM and 6 with DCM. These HCM-linked cTnC mutations include L29Q [1]—A8V, C84Y, and E134D [2]—Q122AfsX30 [3], while the DCM-linked cTnC mutations include E59D/D75Y [4], G159D [5]—Y5H, M103I, D145E, and I148V [6]. cTnC comprises two globular lobes; the amino-(N) and the carboxyl-(C) terminal lobes, which are connected by a flexible linker. The binding of Ca^2+^ to the regulatory site-II of the N-lobe of cTnC is important for triggering structural changes in the regulatory unit (RU), consisting of troponin (Tn) and tropomyosin (Tm).

The binding of Ca^2+^ to cTnC has a strong influence on the rates of transition between *off* and *on* states of RU (Tm-Tn) and the thin filament activation. Moreover, the *on* and *off* kinetic states of RU depend on XB in the force-bearing state through cooperative mechanisms [7, 8]. Therefore, mutations in cTnC lead to the expectation that Ca^2+^ binding kinetics of thin filaments are altered. Two mutations in cTnC are of particular interest to our study: L29Q mutation in the N-lobe, and G159D mutation in the C-lobe. Because of its close proximity to site-II of cTnC, leucine to glutamine substitution at position 29 (L29Q) is considered to have a direct effect on the Ca^2+^ binding properties of cTnC [9–11]. On the other hand, aspartic acid to glycine substitution at position 159 (G159D) is thought to affect interactions of cTnC with cardiac TnI (cTnI) and cardiac troponin T (cTnT), and possibly Tm [12, 13].

The first mutation, L29Q, in cTnC was discovered in a 60-year old male, who was diagnosed with concentric hypertrophy in the left ventricle [1]. However, the patient showed no signs of diastolic and systolic dysfunction [1]. On the other hand, the proband of the G159D *mutation* was diagnosed with DCM at the age of 21 [5]. The proband displayed sudden heart failure symptoms and required a heart transplant two months after diagnosis [5]. Previous *in vitro* studies of L29Q and G159D mutations reported contrasting findings, making it difficult to correlate the functional effects observed to the known cardiac phenotypes. Functional studies on the L29Q mutant reported an increase [10], a decrease [11], or no change in the myofilament Ca^2+^ sensitivity [14, 15]. Studies of G159D mutant also resulted in diverse findings on Ca^2+^ sensitivity; with one group reporting an increase [16], several groups reporting a decrease [14, 17, 18], and other groups reporting no effect at all [19, 20]. Such discrepancies may be primarily related to the use of heterologous tissue/proteins, extracted from pig, bovine, rabbit, human, rat, and mouse species. A cursory look at the proteins from these species reveal multiple amino acid differences, which render it difficult to use them in an assay that is designed to test the effect of a single amino acid exchange. Therefore, to permit an unambiguous understanding of the functional effects of point mutations in cTnC, it is imperative to minimize heterogeneity in experimental conditions.

An important question is whether the effects of cTnC mutations go beyond that of previously observed mild-to-moderate changes in myofilament Ca^2+^ sensitivity [10, 11, 14, 16–18]. Because of the interactions of thin filament kinetics with both Ca^2+^ binding and XB cycling kinetics, we predict that a cTnC mutant-induced effect on the thin filament would affect the other kinetic paradigms; the net effect is a change in the overall mechanodynamics of the whole myofilament system. Therefore, we hypothesized that a cTnC mutant-induced change will be expressed as a change in global myofilament mechanodynamics. To test our hypothesis, we studied the functional effects of L29Q and G159D cTnC mutations on contractile dynamics in detergent-skinned rat cardiac papillary muscle fibers reconstituted with homologous rat cardiac Tn subunits. L29Q substitution caused a small but significant decrease in Ca^2+^ sensitivity, while G159D mutation resulted in no effect. The rate constant that governs the length-mediated XB recruitment dynamics was faster in both L29Q and G159D mutants. The rate constant that describes the length-mediated XB distortion dynamics was faster in the G159D mutant. We discuss these data in terms of cTnC mutant-induced effect on global myofilament mechanodynamics.

## 2. Methods

### 2.1. Preparation of Detergent-Skinned Cardiac Papillary Muscle Fiber Bundles

All animals used in this study received proper care and treatment in accordance with the guidelines set by the Washington State University Institutional Animal Care and Use Committee. Papillary muscle fiber bundles from Sprague-Dawley rat hearts were prepared using the procedure described previously [21]. Briefly, rats were anaesthetized using isoflurane until they were deeply sedated; the depth of anesthesia was confirmed using a pedal withdrawal reflex. Hearts were quickly excised and placed into an ice-cold relaxing solution of pCa 9.0 [22]. Papillary bundles were removed from left ventricles of rat hearts and were further dissected into thinner bundles measuring 2.0-3.0 mm in length and 150–200 *μ*m in cross-section. Thinner fiber bundles were detergent-skinned overnight in relaxing solution that contained 1% Triton-X-100 [23].

### 2.2. Expression and Purification of Recombinant Rat Cardiac Tn Subunits

Recombinant *c-myc* tagged rat cardiac troponin T (*c-myc* RcTnT), troponin I (RcTnI), and troponin C (RcTnC), were all cloned into a pSBETa vector, and were expressed in BL21∗DE3 cells (Novagen, Madison, WI) for protein synthesis. L29Q and G159D substitutions in RcTnC were generated using site-directed DNA mutagenesis techniques and were cloned into a pSBETa vector. BL21∗DE3 cells were lysed and the proteins (*c-myc* RcTnT, RcTnI, and RcTnC) were purified using ion-exchange chromatography techniques, as described previously [22, 24, 25]. In brief, BL21∗DE3 cells of each protein preparation (~4 liters) were spun down and sonicated in 50 mM Tris (pH 8.0 at 4°C), 6 M urea, 5 mM EDTA, 0.2 mM PMSF, 5 mM benzamidine-HCl, 10 mM leupeptin, 1 mM pepstatin, 5 mM bestatin, 2 mM E-64, and 1 mM DTT. The insoluble fraction in each preparation was removed by centrifugation. *c-myc* RcTnT was purified by fractionation of the supernatant from the culture preparation using ammonium sulfate. The pellet from the 70% ammonium sulfate cut was first dissolved in 50 mM Tris (pH 8.0 at 4°C), 6 M urea, 1 mM EDTA, 0.2 mM PMSF, 4 mM benzamidine-HCl, and 1 mM DTT, and then purified by chromatography on a DEAE-fast Sepharose column [22]. RcTnT was eluted from the column using a linear NaCl gradient. RcTnI was purified by directly loading the supernatant from the RcTnI culture preparation onto a CM cation-exchange column and eluted using a linear NaCl gradient. Complete details on cTnI purification can be found in the study by Guo et al. [24]. Wild-type (WT) RcTnC, RcTnC-L29Q, and RcTnC-G159D were purified by loading the supernatant from each RcTnC culture preparation onto a DE-52 anion-exchange column and were eluted using a linear KCl gradient [25]. More details on the purification of cTnC can be found in the study by Pan and Johnson [25]. All fractions containing pure proteins were pooled and dialyzed thoroughly against deionized water containing 15 mM *β*-mercaptoethanol. Dialyzed proteins were lyophilized and stored at −80°C.

### 2.3. Reconstitution of Recombinant Rat Cardiac Tn Subunits into Detergent-Skinned Rat Cardiac Muscle Fiber Bundles

The reconstitution of recombinant cardiac Tn subunits into muscle fiber bundles was performed using a protocol described previously [22]. Briefly, we first prepared an extraction solution containing RcTnT-RcTnI by dissolving *c-myc* RcTnT (1.5 mg/mL, W/V) and RcTnI (1.0 mg/mL, W/V) in 50 mM Tris-HCl (pH 8.0), 6 M urea, 1.0 M KCl, 10 mM DTT, and 0.2 *μ*M PMSF. *c-myc *tagged RcTnT was used in our preparation so that the incorporation of exogenously added Tn could be probed using an antibody against the *c-myc* epitope. Previous studies have shown that presence of 11 amino acid *c*-*myc* epitope at the N-terminus of cTnT has no effect on the normal function of the heart [26–28]. High salt and urea in the extraction solution containing *c-myc* RcTnT-RcTnI were removed by successive dialysis against the following buffers that contain steadily decreasing salt and urea concentrations: 50 mM Tris-HCl (pH 8.0 at 4°C), 4 M urea, 0.7 M KCl, 1 mM DTT, 4 mM benzamidine-HCl, 0.4 mM PMSF, and 0.01% NaN_3_ followed by 50 mM Tris-HCl (pH 8.0 at 4°C), 2 M urea, 0.5 M KCl, 1 mM DTT, 4 mM benzamidine-HCl, 0.4 mM PMSF, and 0.01% NaN_3_. The extraction solution was then extensively dialyzed against the extraction buffer (50 mM BES (pH 7.0 at 20°C), 180 mM KCl, 10 mM BDM, 5 mM EGTA, 6.27 mM MgCl_2_, 1.0 mM DTT, 4 mM benzamidine-HCl, 0.2 mM PMSF, and 0.01% NaN_3_). Detergent-skinned papillary muscle fibers were first treated with this *c-myc* RcTnT + RcTnI protein sample, followed by RcTnC-WT or RcTnC mutant proteins (3 mg/mL) to complete the reconstitution procedure. In our study, detergent-skinned fiber bundles reconstituted with *c-myc* RcTnT + RcTnI + RcTnC-L29Q are referred to as “cTnC_L29Q_ fibers” and those reconstituted with *c-myc* RcTnT + RcTnI + RcTnC-G159D are referred to as “cTnC_G159D_ fibers.” Fiber bundles reconstituted with *c-myc* RcTnT + RcTnI + RcTnC-WT are referred to as “cTnC_WT_ fibers” and served as controls in this study.

### 2.4. SDS-PAGE and Western Blot

We ran 12.5% SDS PAGE to determine the incorporation of cTnC. First, we used 2% SDS solution (10 *μ*L/fiber) to digest the reconstituted fibers for SDS-PAGE, as described previously [29]. SDS-digested fibers were mixed with an equal volume of gel-loading buffer that contained 125 mM Tris-HCl (pH 6.8), 20% glycerol, 2% SDS, 0.01% bromophenol blue, and 50 mM *β*-mercaptoethanol. Digested fibers were run on 12.5% SDS-PAGE to separate the proteins according to their molecular weights [29, 30].

For Western blot analysis, proteins from 12.5% SDS-PAGE were transferred onto a PVDF membrane and cTnC was probed using an anti-TnC primary antibody (Clone M5092922, Fitzgerald Industries International, Concord, MA). The resulting protein profiles from the Western blot were used to assess the incorporation of exogenous cTnC mutants in the reconstituted fibers.

### 2.5. pCa Solutions

For pCa titrations, the fiber was bathed in different pCa solutions ranging from 4.3 to 9.0. The maximal Ca^2+^-activating solution (pCa 4.3) contained the following (in mM concentrations): 50 BES, 5 NaN_3_, 10 phosphoenol pyruvate (PEP), 10 EGTA, 10.11 CaCl_2_, 6.61 MgCl_2_, 5.95 Na_2_ATP, and 31 K-propionate. The relaxing solution (pCa 9.0) contained the following (in mM concentrations): 50 BES, 5 NaN_3_, 10 PEP, 10 EGTA, 0.024 CaCl_2_, 6.87 MgCl_2_, 5.83 Na_2_ATP, and 51.14 K-propionate. In addition, pCa solutions contained the following cocktail of protease inhibitors (in *μ*M concentrations): 10 Leupeptin, 1 Pepstatin, 10 PMSF, 20 A_2_P_5_, and 10 Oligomycin. The reagent concentrations of all pCa solutions were calculated based on a program developed by A. Fabiato and F. Fabiato [31]. The pH of each solution was adjusted to 7.0 using KOH.

### 2.6. Measurements of Isometric Steady-State Force and ATPase Activity

Isometric steady-state force was measured using methods described previously [21, 22]. Briefly, detergent-skinned muscle fiber was attached between a force transducer and a servo motor using aluminum clips and was submerged in a chamber containing pCa 9.0 solution. The baseline SL of the fiber was adjusted to 2.2 *μ*m using laser diffraction technique [32]. The fiber was then activated with a series of pCa solutions starting from pCa 4.3 to 9.0 and the steady-state tension elicited by the fiber in each pCa solution was recorded. These tension values in various pCa solutions were normalized with its respective value in pCa 4.3. The normalized tension values were plotted against pCa to construct pCa-tension relationships for each muscle fiber. All measurements from muscle fibers in this study were performed at 20°C and at a SL of 2.2 *μ*m.

Ca^2+^-activated maximal ATPase activity (pCa 4.3) was measured in reconstituted muscle fibers during isometric steady-state using an assay described previously [28, 33, 34]. In brief, a near UV light was projected through the muscle chamber which was split 50 : 50 for intensity detection at 340 nm and 400 nm wavelengths. Light intensity of the beam at 340 nm was sensitive to NADH, and thus a change in the UV absorbance at 340 nm can be directly correlated to the oxidation of NADH (i.e., ATP usage) through enzymatically coupled reactions [33, 34]. Light intensity of the beam at 400 nm was insensitive to NADH and, therefore, served as the reference signal. An analog divider and log amplifier produced a signal proportional to the amount of ATP consumed (i.e., amount of NADH oxidized) in the muscle chamber solution. After each recording, the UV absorbance signal of NADH was calibrated by multiple rapid injections of 25 pmol of ADP into the bathing solution, with a motor-controlled calibration pipette. Tension cost was estimated by dividing the maximal ATPase activity by the maximal tension in each muscle fiber.

### 2.7. Mechanodynamic Studies

To measure dynamic force-length relationships, we applied sinusoidal muscle length (ML) changes of constant amplitude (±0.5% of ML) to maximally activated muscle fibers [21, 35]. Two chirps, one with frequencies ranging from 0.1 to 4 Hz for a time period of 40 s, and the other with frequencies ranging from 1 to 40 Hz for a time period of 5 s, were administered to emphasize low- and high-frequency force components. The recruitment-distortion (R-D) model was fitted to the overall force response (including both low- and high-frequency components), as described previously [35]. The R-D model predicts a change in muscle force, Δ*F*(*t*), corresponding to a change in muscle length, ΔML(*t*), based on the following equation:

(1) $$\begin{array}{c} \Delta F\left(t\right)=\underset{\text{recruitment}}{\underset{︸}{E_{0}\eta \left(t\right)}}+\underset{\text{distortion}}{\underset{︸}{E_{\infty }x\left(t\right)}}. \end{array}$$

In the equation above, *η*(*t*) and *x*(*t*) are the variables that describe dynamic changes in crossbridge (XB) recruitment and distortion due to changes in ML, respectively. *E*_0_ and *E*_*∞*_ are stiffness coefficients that are proportional to the number of XB in the states, *η*(*t*) and *x*(*t*), respectively.

**Figure 1:**
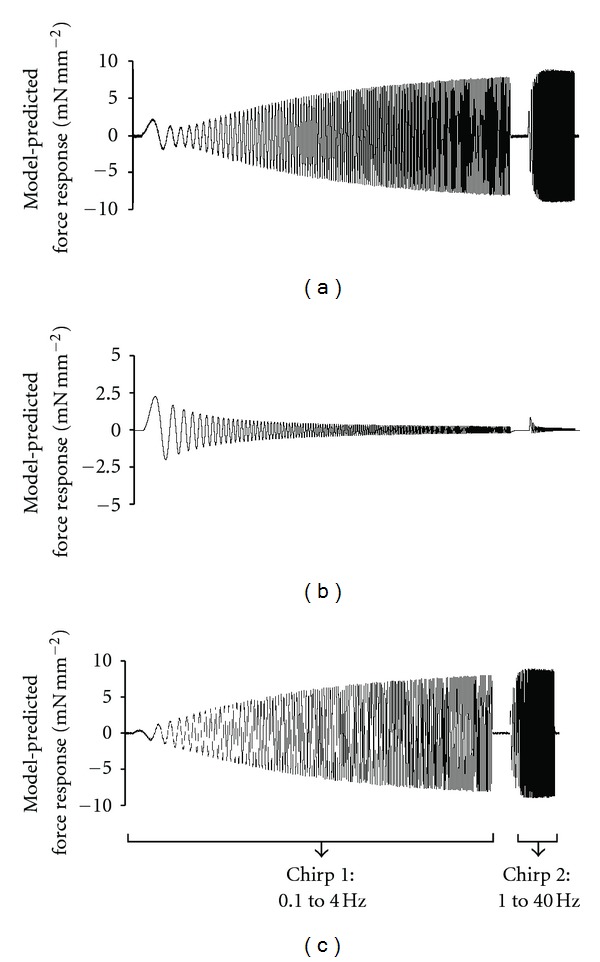
Representative model-predicted force response to chirp-length perturbation for a control-rat fiber (i.e., cTnC_WT_ fiber). (a) Total force response that includes force components due to low-frequency XB recruitment component, *E*_0_*η(*t*), and high-frequency XB distortion component, *E*_∞_*x(*t*). (b) Force response due to low-frequency XB recruitment component, *E*_0_*η(*t*). (c) Force response due to high-frequency XB distortion component, *E*_∞_*x(*t*). *E*_0_ and *E*_*∞*_ represent stiffness magnitudes that scale the contributions of recruitment and distortion components to total stiffness. *η*(*t*) and *x*(*t*) are variables that describe the dynamic changes in the XB recruitment and distortion components due to changes in muscle length. Two chirps were administered to emphasize low- and high-frequency components of the force response: from 0.1 to 4 Hz over 40 s in chirp 1 and from 1 to 40 Hz over 5 s in chirp 2.

**Figure 2:**
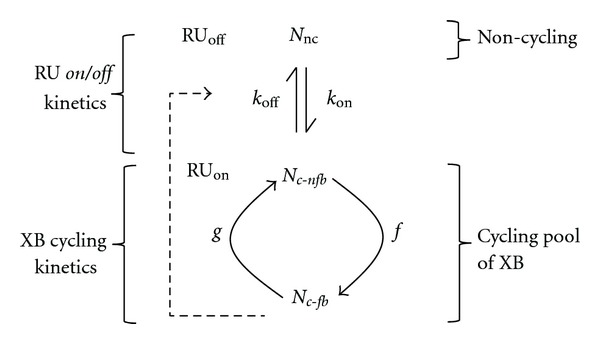
Reduced three-state crossbridge (XB) model scheme depicting regulatory unit (RU; Tm-Tn) kinetics and XB cycling kinetics. This scheme is adapted from Campbell [36]. *k*_*on*⁡_ and *k*_*off*⁡_, represent the RU *on/off* rates and are functions of Ca^2+^ bound to cTnC. Once turned on by the binding Ca^2+^, RU permits the transition of XB from the non-cycling (*N*_nc_) pool to the cycling pool of XB. The cycling pool of XB includes two states of XB: cycling non-force-bearing (*N*_*c*-nfb_) and cycling force-bearing (*N*_*c*-fb_). The transition between non-cycling and cycling pools is mainly regulated by *k*_*on*⁡_/*k*_*off*⁡_ kinetics of RU. The influence of the force-bearing XB on the RU *on/off* kinetics is represented by the feedback arrow (dashed line). *f* and *g* represent the rate constants governing forward transition, *N*_*c*-nfb_ → *N*_*c*-fb_, and backward transition, *N*_*c*-fb_ → *N*_*c*-nfb_, respectively.

The R-D model was fitted to the total force response elicited by the fiber with ΔML(*t*) as the input to estimate four important model parameters—*E*_0_, *b*, *E*_*∞*_, *c* [35]. Previously, we showed that a big advantage of the R-D model is that the total force response (Figure 1(a)) could be uniquely separated into two components: (1) force response due to low-frequency recruitment component (*b*, *E*_0_; Figure 1(b)) and (2) force response due to high-frequency distortion component (*c*, *E*_*∞*_; Figure 1(c)). This feature of the R-D model was successfully used previously to elicit the dynamic features of the respective force components in constantly activated muscle fibers [21, 35].

### 2.8. Crossbridge Model Scheme

In this study, we interpreted our experimental data using a reduced three-state model, as illustrated in Figure 2 [36]. In brief, the model describes thin filament activation using three kinetic processes:

1. Ca^2+^ binding to the thin-filament regulatory unit (RU; Tm-Tn);
2. RU switching between *on* and *off* states;
3. XB cycling between attached and detached states.

To describe these respective processes, the total XB population is subdivided into two pools: a non-cycling pool (*N*_nc_) and a cycling pool (*N*_*c*-nfb_ and *N*_*c*-fb_). The kinetic processes 1 and 2 are lumped into a single kinetic step that represents RU *on*/*off* kinetics. The *on*/*off* kinetics of RU are strongly affected by the interactions of thin filament regulatory processes with Ca^2+^ binding/dissociation kinetics of cTnC. The preferred state of the RU is “*off*” when Ca^2+^ is not bound to cTnC, whereas, the preferred state is “*on*” when Ca^2+^ is bound to cTnC. When RU are *off* (i.e., no activator Ca^2+^), the interactions of myosin head with actin are inhibited due to the steric blocking of actin sites by RU. In this scenario, all XB will populate in the non-cycling *N*_nc_ state. The binding of Ca^2+^ to cTnC will switch the RU *on* by removing their steric blocking effect on actin sites, favoring the entry of XB into the cycling, *N*_*c*-nfb_ and *N*_*c*-fb_, states. The rate at which the XB transition from non-cycling to cycling pool (i.e., RU *on* kinetics) is represented by *k*_*on*⁡_, whereas, the rate at which XB transition from cycling to non-cycling pool (i.e., RU *off* kinetics) is represented by *k*_*off*⁡_. It is important to note that *k*_*on*⁡_ and *k*_*off*⁡_ are affected by both Ca^2+^ binding/dissociation kinetics and force-bearing XB. XB within the cycling pool may exist in two distinct states, a non-force-bearing state, *N*_*c*-nfb_, and a force-bearing state, *N*_*c*-fb_. In the cycling pool, XB alternate between *N*_*c*-nfb_ and *N*_*c*-fb_ states according to the rate constants, *f* and *g*.

In the context of this reduced three-state XB model scheme, the R-D model parameters—*E*_0_, *b*, *E*_*∞*_, and *c*—specifically represent the following: *E*_*∞*_ is the magnitude of instantaneous length-mediated increase in stiffness due to the rapid distortion of XB in the *N*_*c*-fb_ state; *E*_0_ is the magnitude of length-mediated increase in stiffness caused by an increase in the number of XB in the *N*_*c*-fb_ state; recruitment rate constant, *b* incorporates various length-sensing mechanisms including thin filament overlap, XB attachment, and its amplification by cooperativity (indicated by dashed arrow in Figure 2). In other words, the RU *on*/*off* rates, *k*_*on*⁡_ and *k*_*off*⁡_, and XB cycling kinetics defined by rate constants, *f* and *g*, all coalesce into a single rate constant, *b*. Parameter *c* governs the distortion dynamics and has a strong dependence on the XB detachment rate, *g* [35].

### 2.9. Data Analysis

Data are shown as mean ± SEM. pCa required to elicit half maximal tension, *pCa*_50_, and the Hill coefficient, *n*_H_, were estimated by fitting the Hill's equation to normalized tension data. Our data included three groups: cTnC_WT_, cTnC_L29Q_, and cTnC_G159D_ fibers. Statistical differences between cTnC_WT_,  cTnC_L29Q_, and cTnC_G159D_ fibers were analyzed using one-way ANOVA. Minimal statistical significance was set at *α* = 0.05.

## 3. Results

### 3.1. Incorporation of Recombinant Mutant cTnC Proteins into Myofibers

**Figure 3:**
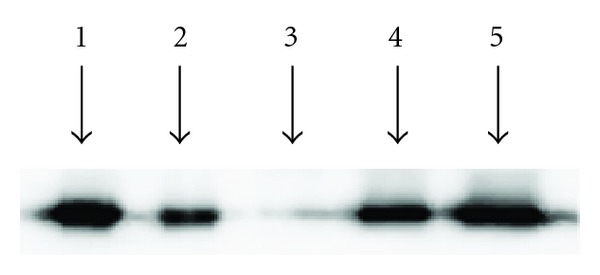
Western blot analysis of detergent-skinned rat cardiac muscle fibers reconstituted with cTnC_WT_, cTnC_L29Q_ or cTnC_G159D_. Muscle protein samples were separated on 12.5% SDS-PAGE. Proteins from gel were transferred to a PVDF membrane and RcTnC was probed with an anti-TnC primary antibody (Fitzgerald M5092922). Protein profiles in *lanes* 1–5 represent the following: *lane* 1, purified RcTnC; *lane* 2, fibers reconstituted with *c-myc* RcTnT + RcTnI + RcTnC-WT; *lane* 3, fibers reconstituted with *c-myc* RcTnT + RcTnI; *lane* 4, fibers reconstituted with *c-myc* RcTnT + RcTnI + RcTnC-L29Q; *lane* 5, fibers reconstituted with *c-myc* RcTnT + RcTnI + RcTnC-G159D.

Figure 3 shows the cTnC protein profiles from various reconstituted muscle fiber groups. The Western blot against anti-cTnC primary antibody confirmed the absence of native cTnC in the cTnT-cTnI treated fiber bundles, clearly demonstrating a near-complete removal of endogenous Tn units using our exchange procedure (Figure 3; *lane* 3). Furthermore, the Western blot confirmed that the recombinant cTnC_L29Q_ (*lane* 4) and cTnC_G159D_ (*lane* 5) mutants were incorporated properly in the reconstituted fiber bundles.

### 3.2. Effect of cTnC_L29Q_ and cTnC_G159D_ on Ca^2+^-Activated Maximal Tension, Maximal ATPase Activity, and the Magnitude of Myofiber Dynamic Stiffness

We first assessed the effects of cTnC_L29Q_ and cTnC_G159D_ mutants on Ca^2+^-activated maximal tension and maximal ATPase activity. To determine the maximal tension, reconstituted fibers were bathed in pCa 4.3 solution until they reached a steady-state isometric force. The isometric steady-state force was then converted to tension by expressing it as force per cross-sectional area. The Ca^2+^-activated maximal tension values (in mN·mm^−2^) in cTnC_WT_, cTnC_L29Q_, and cTnC_G159D_ fibers were 57 ± 2 (*n* = 15), 56 ± 2 (*n* = 10), and 53 ± 2 (*n* = 14), respectively. These data demonstrate that the Ca^2+^-activated maximal tension values in cTnC_L29Q_- and cTnC_G159D_-reconstituted fibers were similar to that of cTnC_WT_-reconstituted fibers. Thus, both cTnC_L29Q_ and cTnC_G159D_ had no impact on Ca^2+^-activated maximal tension. Our observations in maximal tension agree well with many previous *in vitro* studies of L29Q [10, 14, 15] and G159D [14, 16, 19, 20] mutations, confirming that both these mutants did not affect the maximal tension. Thus, our tension data further substantiated our conclusion from the Western blot (Figure 3) that the reconstitution of cTnC_L29Q_ and cTnC_G159D_ mutants into detergent-skinned fibers was normal.

We also measured the maximal ATPase activity in reconstituted muscle fibers using a procedure described in Methods. The Ca^2+^-activated maximal ATPase values (in pmol · mm^−3^ · s^−1^) in cTnC_WT_, cTnC_L29Q_, and cTnC_G159D_ fibers were 193 ± 9 (*n* = 9), 187 ± 8 (*n* = 8), and 219 ± 13 (*n* = 9), respectively. Although the maximal ATPase activity was not significantly different between various groups, cTnC_G159D_ fibers demonstrated an increasing trend in the maximal ATPase consumption (by 13.4%) when compared to that of cTnC_WT_ fibers.

We also estimated the magnitudes of XB distortion dynamics (*E*_*∞*_) and recruitment dynamics (*E*_0_) in cTnC_WT_-,  cTnC_L29Q_-, and cTnC_G159D_-reconstituted fibers. In previous studies, we have demonstrated that *E*_*∞*_ is a measure of the number of strongly-bound XB and *E*_0_ is a measure of the number of newly-recruited XB due to an increase in muscle length [22]. Both *E*_0_ and *E*_*∞*_ estimates in cTnC_L29Q_- and cTnC_G159D_-reconstituted fibers were not significantly different from those of cTnC_WT_-reconstituted fibers. *E*_0_ estimates (in mN mm^−3^) in cTnC_WT_, cTnC_L29Q_, and cTnC_G159D_ fibers were 173 ± 10 (*n* = 15), 151 ± 10 (*n* = 10), and 148 ± 11 (*n* = 14), respectively. The corresponding *E*_*∞*_ estimates (in mN mm^−3^) were 786 ± 32 (*n* = 15), 750 ± 47 (*n* = 10), and 788 ± 43 (*n* = 14), respectively. Thus, both cTnC_L29Q_ and cTnC_G159D_ had no effect on the number of strongly-bound XB and the number of newly-recruited XB due to a change in muscle length. Collectively, our results from the Western blot, Ca^2+^-activated maximal tension, and the magnitude of XB distortion dynamics indicate that both cTnC mutants incorporated properly into the myofibers and that they had no effect on either the number of strongly-bound XB or maximal tension.

### 3.3. Effect of cTnC_L29Q_ and cTnC_G159D_ on Myofilament Ca^2+^ Sensitivity and Cooperativity

**Figure 4:**
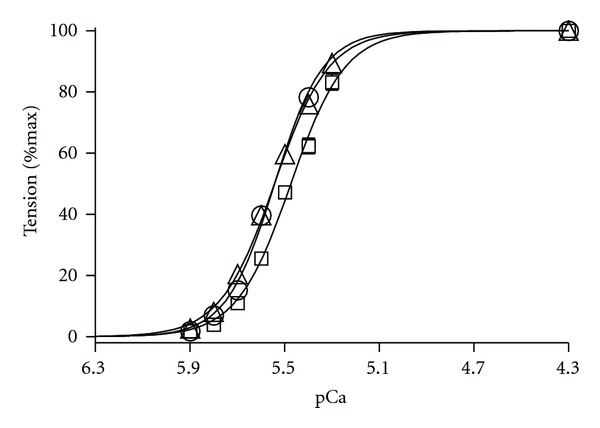
Comparison of normalized pCa-tension relationships in detergent-skinned fibers reconstituted with cTnC_WT_, cTnC_L29Q_, or cTnC_G159D_. Isometric steady-state tensions elicited by each fiber in various pCa solutions were normalized with its respective value in pCa 4.3 solution. The normalized tensions were plotted against pCa to construct the pCa-tension relationship. The Hill equation was fitted to the normalized pCa-tension relationships to estimate myofilament Ca^2+^ sensitivity (pCa_50_) and the Hill coefficient (*n*_H_). *pCa*_50_ and *n*_H_ values are shown in Figures 5(a) and 5(b), respectively. The curves presented here are Hill fits to pCa-tension relationships in cTnC_WT_ (○), cTnC_L29Q_ (□), and cTnC_G159D_ (∆) fibers, respectively. Values are expressed as mean ± SEM. Number of fibers tested in each group is as follows: cTnC_WT_, *n* = 15; cTnC_L29Q_, *n* = 10; cTnC_G159D_, *n* = 14.

**Figure 5:**
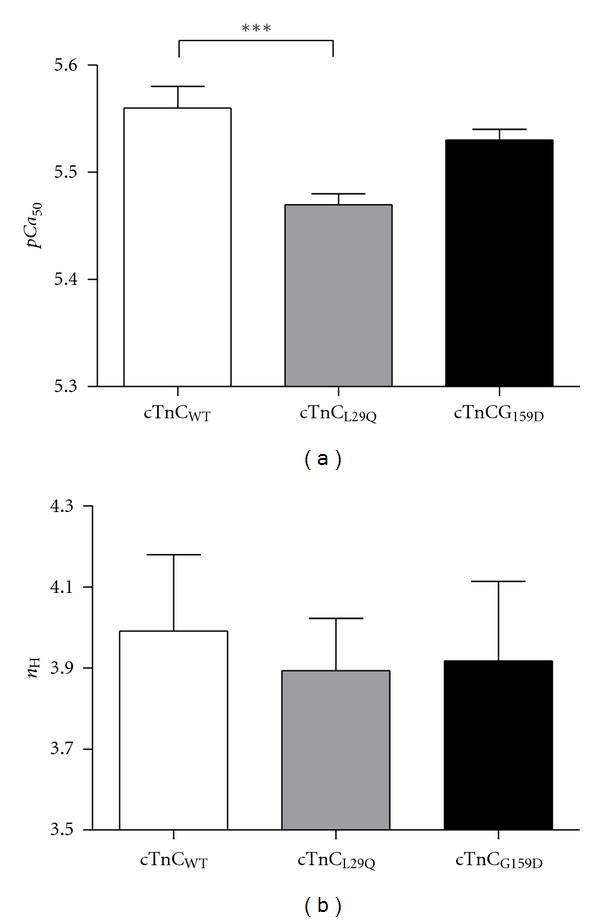
Comparison of myofilament Ca^2+^ sensitivity (*pCa*_50_) and cooperativity (*n*_H_) of pCa-tension relationships in detergent-skinned fibers reconstituted with cTnC_WT_, ccTnC_L29Q_, or cTnC_G159D_. (a) Effects of RcTnC mutants on *pCa*_50_ (b) Effects of RcTnC mutants on *n*_H_. The Hill equation was fitted to the normalized pCa-tension relationships to estimate *pCa*_50_ and *n*_H_. One-way ANOVA was used to compare *pCa*_50_ and *n*_H_ estimates in cTnC_L29Q_ and cTnC_G159D_ fibers with the data from cTnC_WT_ fibers as controls. Values are expressed as mean ± SEM. Number of fibers tested in each group is as follows: cTnC_WT_, *n* = 15; cTnC_L29Q_, *n* = 10; cTnC_G159D_, *n* = 14. Minimal statistical significance was set at *α* = 0.05. ***P < 0.001.

To determine if cTnC_L29Q_ and cTnC_G159D_ mutants altered myofilament Ca^2+^ sensitivity (*pCa*_50_) and cooperativity (*n*_H_), we fitted the Hill's equation to normalized tension data obtained at different pCa.  Figure 4 illustrates a comparison of pCa-tension relationships between cTnC_WT_, cTnC_L29Q_, and cTnC_G159D_ fibers. When compared to cTnC_WT_ fibers, cTnC_L29Q_ fibers showed a small but significant decrease in *pCa*_50_, as indicated by a rightward shift in the pCa-tension relationships (*P* < 0.001; Figures 4 and 5(a)). However, estimates of *pCa*_50_ in cTnC_G159D_ fibers were not different from those of cTnC_WT_-reconstituted fibers (Figures 4 and 5(a)), suggesting that cTnC_G159D_ did not affect myofilament Ca^2+^ sensitivity. These data demonstrate that cTnC_L29Q_ and cTnC_G159D_ behave differently, with respect to their effect on myofilament Ca^2+^ sensitivity. *n*_H_ was unaltered in fibers reconstituted with either cTnC_L29Q_ or cTnC_G159D_, suggesting that the myofilament cooperativity was unaffected by both cTnC mutants (Figure 5(b)).

### 3.4. Effect of cTnC_L29Q_ and cTnC_G159D_ on XB Detachment Kinetics

**Figure 6:**
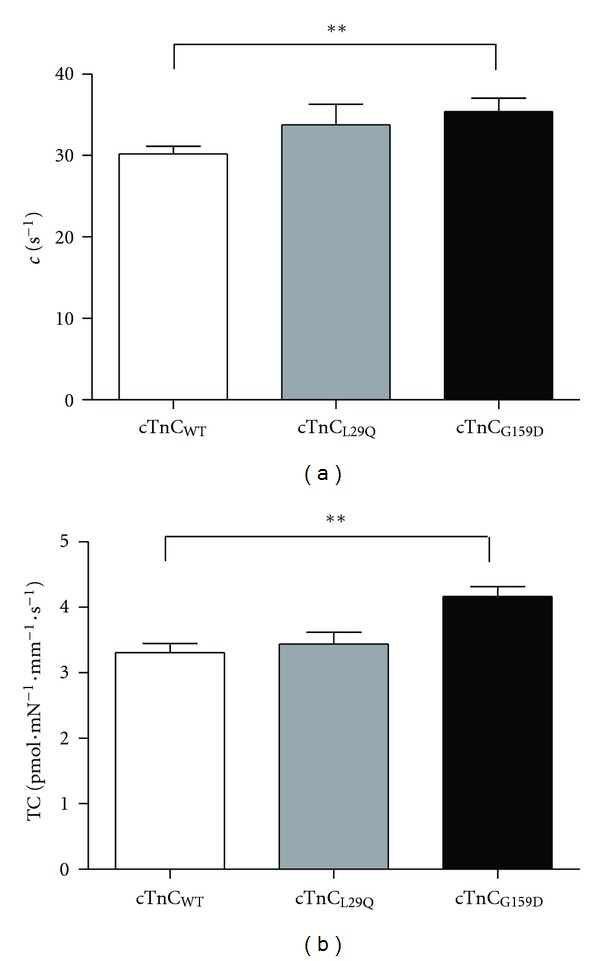
Comparison of XB distortion rate constant, *c*, and tension cost, TC, in detergent-skinned fibers reconstituted with cTnC_WT_, cTnC_L29Q_, or cTnC_G159D_. (a) Effects of RcTnC mutants on *c. c* was estimated by fitting the R-D model to the force responses from muscle fibers to chirp-length perturbations [35]. (b) Effects of RcTnC mutants on TC. TC was estimated by dividing the maximal ATPase activity with the maximal tension elicited by the muscle fiber. One-way ANOVA was used to compare estimates of *c* and TC in cTnC_L29Q_ and cTnC_G159D_ fibers with the data from cTnC_WT_ fibers as controls. Values are reported as mean ± SEM. Number of fibers tested in each group is as follows: cTnC_WT_, *n* = 15; cTnC_L29Q_, *n* = 10; cTnC_G159D_, *n* = 14. Minimal statistical significance was set at *α* = 0.05, **P < 0.01.

Myofilament Ca^2+^ sensitivity may also be affected by changes in XB detachment kinetics, *g*. For example, an increase in *g* may also decrease myofilament Ca^2+^ sensitivity. To examine whether cTnC mutations affected the XB detachment kinetics, we estimated length-mediated XB distortion dynamic, *c*, and tension cost, TC, in detergent-skinned muscle bundles reconstituted with cTnC_WT_,  cTnC_L29Q_, or cTnC_G159D_ mutants. Previously, we have shown that TC is strongly correlated to *c* and that both have a strong dependence on the XB detachment rate, *g* [35]. Thus, changes in *c* and TC may convey important effects of cTnC_L29Q_ and cTnC_G159D_ on XB detachment kinetics. Our estimates of *c* and TC in cTnC_L29Q_ fibers were not significantly different from those of cTnC_WT_ fibers (Figures 6(a) and 6(b), resp.). However, estimates of *c* and TC in cTnC_G159D_ fibers were significantly higher by 15% (*P* < 0.01; Figure 6(a)) and 26% (*P* < 0.01; Figure 6(b)), respectively, suggesting that the cTnC_G159D_ mutant increased XB detachment rate. It is important to note that although the maximal tension and maximal ATPase activity of cTnC_G159D_ fibers were not significantly different from those of cTnC_WT_ fibers, the TC was significantly higher. The reason for this is that the maximal tension is slightly lower (by 7%) and the maximal ATPase is slightly higher (by 13.4%), making the TC significantly higher in cTnC_G159D_ fibers when compared to that of cTnC_WT_ fibers.

### 3.5. Effect of cTnC_L29Q_ and cTnC_G159D_ on the Rate of XB Recruitment Dynamics

**Figure 7:**
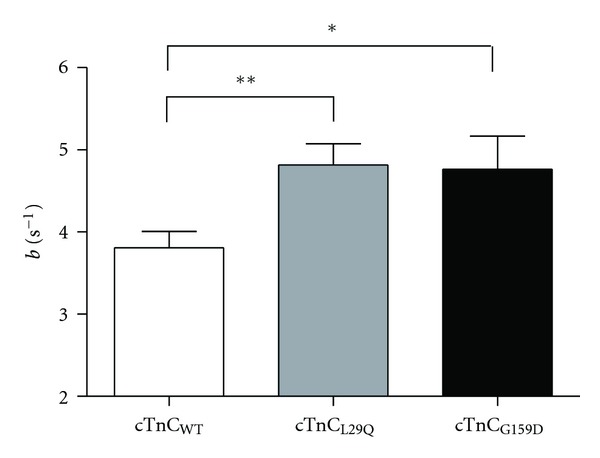
Comparison of XB recruitment dynamic, *b*, in detergent-skinned fibers reconstituted with cTnC_WT_, cTnC_L29Q_, or cTnC_G159D_. *b* was estimated by fitting the R-D model to the force responses from muscle fibers to chirp-length perturbations [35]. One-way ANOVA was used to compare estimates of *b* in cTnC_L29Q_ and cTnC_G159D_ fibers with the data from cTnC_WT_ fibers as controls. Values are expressed as mean ± SEM. Number of fibers tested in each group is as follows: cTnC_WT_, *n* = 15; cTnC_L29Q_, *n* = 10; cTnC_G159D_, *n* = 14. Minimal statistical significance was set at *α* = 0.05. *P < 0.05; **P < 0.01

cTnC mutant-induced effect on myofilament Ca^2+^ sensitivity may indicate an effect on RU *on*/*off* kinetics. Because changes in RU *on*/*off* kinetics have an impact on the rate of XB recruitment dynamics, we measured the rate constant of XB recruitment dynamics, *b*, using dynamic muscle fiber stiffness measurements in cTnC_WT_-,  cTnC_L29Q_-, and cTnC_G159D_-reconstituted fibers. As illustrated in Figure 7, our observations show that *b* speeds by 26.5% (*P* < 0.01) in cTnC_L29Q_ and by 25.3% (*P* < 0.05) in cTnC_G159D_ fibers. Therefore, our data suggest that both cTnC_L29Q_ and cTnC_G159D_ mutants affect thin filament processes that mediate the length-dependent effects on the rate of XB recruitment dynamics.

## 4. Discussion

Experiments presented here provide new evidence for the mechanism by which TnC mutations bring about global change in myofilament mechanodynamics. To our knowledge, this is the first study that addresses important unresolved questions. (1) What is the effect of TnC mutations on XB recruitment and distortion dynamics? (2) How does the interplay between Ca^2+^ binding kinetics and XB cycling kinetics produce a global change in myofilament mechanodynamics? Global myofilament kinetics is governed by interactions between Ca^2+^ binding kinetics and XB cycling kinetics [7, 8, 36]. We have tested this interplay effect by measuring pCa-tension relationship and myofiber dynamic stiffness in rat cardiac muscle fibers reconstituted with L29Q and G159D cTnC mutants. New data from our study provides a mechanistic basis for the functional effects observed in humans containing L29Q and G159D mutations in cTnC.

 Our finding that the L29Q mutation elicited a small but significant decrease in myofilament Ca^2+^ sensitivity, *pCa*_50_ (~0.09 units; Figure 5(a)), is consistent with the report from a previous study [11]. Furthermore, our observation that *pCa*_50_ remained unaltered by the G159D mutation is also in agreement with previous studies which employed reconstituted assays [19, 20]. However, there are significant discrepancies between our observations and other studies, which reported contrasting findings on *pCa*_50_ for either L29Q [10, 14, 15] or G159D mutation [14, 16–18]. Some of these discrepancies between our study and others [10, 14–18] may be attributed to many experimental variants, including but not limited to the type of proteins used (i.e., homologous or heterologous), reconstitution techniques employed, phosphorylation status of cTnI in the reconstituted system, species-specific differences (mouse, rat, pig, rabbit, human), and so forth.

Our study showed a decrease in myofilament Ca^2+^ sensitivity with the L29Q mutation, while a previous study showed no effect [15]. On the other hand, our finding that G159D mutation had no effect on Ca^2+^ sensitivity is in contrast with two previous studies which showed a decrease [17, 18]. Such discrepancies between our study and the aforementioned studies may be likely due to the use of heterologous proteins. For example, while Neulen et al. [15] reconstituted human cardiac Tn subunits into mouse cardiac myofilaments, Mirza et al. [17] and Robinson et al. [18] used rabbit skeletal (F-actin and myosin) and human cardiac (TnC/I/T and Tm) muscle proteins in their *in vitro* ATPase assays. The use of such a heterologous reconstituted system to understand the functional effect of a single site mutation in cTnC makes it difficult to ascribe the findings directly to the specific substitution introduced. Our study avoids this issue through reconstitution of rat papillary muscle fibers with homologous recombinant rat cardiac Tn subunits. 

The method used for reconstituting the recombinant proteins into the thin filament may also play a role in such discrepancies. For example, two previous investigations used CDTA treatment to selectively extract the endogenous cTnC subunits in their experimental preparations and to reconstitute them with recombinant cTnC mutants [14, 15]. In this regard, our study differs in that we removed all endogenous cardiac Tn subunits from rat papillary muscle fibers and reconstituted them with recombinant rat cardiac Tn subunits. Furthermore, because Dweck et al. [14] and Neulen et al. [15] confined the extraction and reconstitution to cTnC in their studies, it may be possible that phosphorylation of endogenous cTnI in their experimental preparations might be different from our preparations (reconstituted with nonphosphorylated cTnI). In addition, functional effects can also be attributed to the use of lower rodents (rats in our study) versus larger animals (pigs in the study by Dweck et al. [14] and humans in the study by Dyer et al. [16]). These possible factors may likely explain the discrepancies observed between our study and the aforementioned studies of L29Q and G159D mutations.

The first question that needs to be addressed in our study is, “how does the L29Q mutation brings about a small change in Ca^2+^ sensitivity?” The L29Q mutation may affect myofilament Ca^2+^ sensitivity via either a direct effect on Ca^2+^ binding to site-II of TnC or an indirect allosteric effect on the overall configuration of the regulatory unit (RU; Tn-Tm). Evidence pertaining to these claims comes from previous Ca^2+^ binding affinity studies of L29Q mutation, which suggest a possible L29Q-induced destabilization of helix A in cTnC. This effect of L29Q on helix A may affect the Ca^2+^ binding properties at site II [10] and/or the interaction between cTnC and cTnI [11]. Regardless of the way the L29Q mutation affects myofilament Ca^2+^ sensitivity, we expect that the kinetics of *on*/*off* transition of the RU will be affected. Based on the XB model scheme shown in Figure 2, we think that a decrease in Ca^2+^ sensitivity may be linked to RU *on*/*off* kinetics, *k*_*on*⁡_ and *k*_*off*⁡_. Because force-bearing XB have an effect on *on*/*off* kinetics of RU, we first established that the infinite frequency stiffness (*E*_*∞*_) was not altered in L29Q-reconstituted fibers. Previously, we have demonstrated that *E*_*∞*_ and Ca^2+^-activated maximal tension (*T*_max⁡_) are both measures of the number of parallel force-bearing XB [35]. Our observations that *E*_*∞*_ and *T*_max⁡_ were unaltered suggested that the L29Q mutation did not affect the number of force-bearing XB. Another mechanism that may decrease myofilament Ca^2+^ sensitivity is through an augmenting effect on XB detachment rate, *g*. Our observation that both the rate constant for XB distortion dynamics (*c*) and tension cost (TC) were unaltered suggested that the L29Q mutation did not affect *g* (Figures 6(a) and 6(b), resp.). Our finding is consistent with a previous study which showed that the velocity of unloaded shortening was unaltered by the L29Q mutation [15]. Collectively, these observations suggest that the L29Q mutation may affect the equilibrium between the non-cycling and cycling XB pools by affecting the *k*_*on*⁡_/*k*_*off*⁡_ of RU (Figure 2).

An impact on *k*_*on*⁡_/*k*_*off*⁡_ of RU will affect the dynamics of XB recruitment [22, 35]. To determine the effects on XB dynamics, we assessed the length-mediated effects on XB recruitment rate, *b*, in L29Q-reconstituted fibers. *b* increased by 26.5% in L29Q-reconstituted fibers. It is important to note that dynamics of XB recruitment are affected by various length-sensing mechanisms, including changes in the thin filament overlap and XB cycling kinetics [35]. In addition, XB themselves affect the balance between RU *on*/*off* states via cooperative mechanisms (dashed line with feedback arrow in Figure 2). Therefore, *b* is a function of the RU *on*/*off* rates, *k*_*on*⁡_ and *k*_*off*⁡_, as well as the rate parameters that define XB cycling kinetics, *f* and *g* [35]. Because *g* was unaffected by the L29Q mutation, an increase in *b* may be associated with an increase in any of the following rate constants—*k*_*off*⁡_, *k*_*on*⁡_, and *f* (Figure 2). However, an increase in *k*_*on*⁡_ or *f* is unlikely because such effects would increase the number of force-bearing XB and the *T*_max⁡_, effects that are not observed in L29Q-reconstituted fibers. Thus, we predict that an increase in *b* in L29Q-reconstituted fibers is probably due to an increase in the RU *off* rate, *k*_*off*⁡_. Because *k*_*on*⁡_ and *k*_*off*⁡_ are functions of Ca^2+^ bound to RU, an increase in *k*_*off*⁡_ is consistent with a decrease in *pCa*_50_ observed in L29Q-reconstituted fibers. A higher *k*_*off*⁡_ stabilizes XB in the *N*_nc_ state, thereby reducing their transitioning into the cycling pool, *N*_*c*-nfb_ (Figure 2). Because the population of XB in the cycling pool depends on the net effect of *k*_*on*⁡_ and *k*_*off*⁡_, an increase in *k*_*off*⁡_ acts to reduce XB recruitment, resulting in a lower tension in L29Q fibers at submaximal Ca^2+^ activations. However, at maximal Ca^2+^ activation, all the troponin units have near-maximal Ca^2+^ bound to TnC, thereby increasing the probability of more force-bearing XB (i.e., *f*). Thus, the net effect of *k*_*off*⁡_ on *f* will be minimal at maximal Ca^2+^-activation, resulting in no effect on the number of force-bearing XB and the maximal force.

The G159D mutation had no effect on *pCa*_50_, *T*_max⁡_, and *E*_*∞*_. Thus, its effect on the heart must come from another source of primary myofilament defect that remains poorly understood. Our measurements in G159D-reconstituted fibers showed that both the rate constant for XB distortion dynamic, *c*, and TC were significantly higher in G159D fibers (Figures 6(a) and 6(b)). Because both *c* and TC have a strong dependence on *g* [21, 35], higher *c* and TC are indicative of an increase in the rate of XB detachment in G159D fibers. In addition, G159D fibers also demonstrated a significant increase in the rate constant for XB recruitment dynamic, *b* (Figure 7). As discussed before, an increase in *b* may be associated with an increase in any of the rate constants—*k*_*on*⁡_, *k*_*off*⁡_, *f*, and *g*. However, an unaltered *pCa*_50_ supports the notion that the RU *on*/*off* rates, *k*_*on*⁡_ and *k*_*off*⁡_, are not affected by the G159D mutation. Therefore, an increase in *b* may be attributed to an increase in *g* alone or an increase in both *f* and *g*. An increase in *g* alone is unlikely because such an effect would reduce the number of force-bearing XB, which would decrease both *T*_max⁡_ and myofilament Ca^2+^ sensitivity. Therefore, a plausible interpretation is that the G159D mutant may increase both *f* and *g*, causing XB to cycle faster. Although conjectural, the following explanation may shed some light on how the G159D mutation affects the dynamics of myofilament activation. The location of the mutant in the C-domain of cTnC suggests that it is unlikely to have a direct impact on Ca^2+^-mediated regulatory steps. One possible mechanism by which a mutation in the C-domain of cTnC may affect XB recruitment dynamics is by altering the dynamics of XB-mediated effects on RU. Because part of the C-domain (adjacent to G159D) is in close contact with the IT arm of cardiac Tn [13, 37], it is possible that this mutation affects the global configuration of the IT arm in such a way that the status of RU is affected. The IT arm is formed by several key interactions, including that between cTnT and cTnI and hydrophobic interactions between cTnI and the C-domain of cTnC. Thus, it is reasonable to postulate that the IT arm of the troponin complex is affected by the G159D mutation.

Our study adds the following new data to our existing knowledge on the L29Q and G159D mutant-cardiac phenotype relationships. The L29Q mutation caused a small decrease in myofilament Ca^2+^ sensitivity, which was linked to an increase in RU *off *rate. Such an effect can be linked to the cardiac phenotype (i.e., hypertrophy) in L29Q proband by considering the fact that cardiac muscle cells operate under a very narrow range of physiological [Ca^2+^]_free_. Therefore, any significant decrease in myofilament Ca^2+^ sensitivity may cause a significant decrease in tension at that given [Ca^2+^]_free_. Thus, the hypertrophy associated with the L29Q substitution may be a compensatory response to overcome a decrease in tension caused by an attenuation of myofilament Ca^2+^ sensitivity. On the other hand, G159D mutation resulted in an increase in the XB cycling rate that is, *f* and *g*, with no effects on maximal tension and Ca^2+^ sensitivity. Such increases in the XB cycling rate causes an increase in the cost of tension maintenance, subjecting the heart to a chronic stress to meet this increased energy demand. This functional consequence may be an impetus for the ventricular dilatation associated with the G159D mutation.
